# Decalcification prevention around orthodontic brackets bonded to bleached enamel using different topical agents

**DOI:** 10.1186/s40510-017-0170-4

**Published:** 2017-06-12

**Authors:** Ferial Ahmed Msallam, Mohammed El-Awady Grawish, Ahmad Mohammed Hafez, Yasser Lotfy Abdelnaby

**Affiliations:** 1 Department of Orthodontics , Faculty of Dentistry, Tripoli University, Tripoli, Libya; 2 Department of Orthodontic, Faculty of Dentistry, Mansoura University, Mansoura, Egypt; 3 Department of Oral Biology, Faculty of Dentistry, Mansoura University, Mansoura, Egypt; 4 Department of Orthodontic, Faculty of Dentistry, Mansoura University, Mansoura, Egypt

**Keywords:** Decalcification, Laser fluorescence, Bleached enamel

## Abstract

**Background:**

The present study was conducted to evaluate the effect of different topical agents utilized for prevention of enamel decalcification around orthodontic brackets bonded to bleached and non-bleached enamel.

**Methods:**

Human maxillary premolars (*n* = 120) were divided into two equal groups. Teeth in group I were left without bleaching while those in group II were bleached with Vivastyle gel. Metal brackets were bonded to all the teeth using light-cured adhesive. Each group was divided into six equal subgroups (A, B, C, D, E, and F). In subgroup A, no material was applied (control). In subgroups B, C, D, E, and F, the following materials were applied respectively: Profluorid varnish, Enamel Pro Varnish, Ortho-Choice Ortho-Coat, GC Tooth Mousse, and GC MI Paste Plus. All teeth were cycled in a demineralization solution/artificial saliva for 15 days. Laser fluorescence was used to measure the level of enamel mineralization. The data were statistically analyzed.

**Results:**

Regarding the non-bleaching subgroups, all studied material revealed significant demineralization reduction in comparison to the control subgroup (*P* < 0.05). Ortho-Choice Ortho-Coat revealed the highest significant effect while GC Tooth Mousse showed the least effect. In bleached subgroups, Profluorid varnish, Enamel Pro Varnish, and Ortho-Choice Ortho-Coat significantly reduced demineralization (*P* < 0.05) while either GC MI Paste Plus or GC Tooth Mousse had no significant effects (*P* > 0.05).

**Conclusions:**

Ortho-Choice Ortho-Coat, and Profluorid and Enamel Pro varnishes could be utilized successfully to reduce enamel demineralization around brackets bonded to either bleached or non-bleached enamel. GC MI Paste Plus and GC Tooth Mousse were effective only in non-bleached enamel.

## Background

White spot lesions (WSLs) considered one of the deleterious effects associated with the orthodontic treatments. It could negatively affect the satisfaction of either the orthodontists or the patients toward the treatment outcomes regardless its quality [1].

The fixed orthodontic appliances act as food stagnation area and increase the potential of development of dental plaque. The levels of acidogenic bacteria become significantly elevated. These bacteria will work on fermentable carbohydrates resulting in the production of acid by-products as well as lowering the pH of the plaque. Finally, when the pH decreases below the threshold for remineralization, carious decalcification occurs [2, 3].

Several methods have been used for prevention of WSLs. Patient motivation and oral hygiene instruction, as well as home use of fluoride supplements such as fluoride rinses or gels have been demonstrated to be effective [4]. Unfortunately, this approach is totally dependent upon unpredictable patient compliance [5]. On the other hand, non-compliance or professional methods of fluoride application were used that include the utilization of fluoride varnish, sealant or coat, fluoride-released adhesive, and fluoride-released elastomeric [6–8]. However, this approach requires complex operative procedures.

Many types of fluoride varnishes of different compositions and concentrations were developed. Sodium fluoride (NaF) varnish is one of the common fluoride varnishes [9]. Its action depends on the formation of calcium fluoride (CaF_2_). In addition, it provides fluoride reservoir on the enamel surface against cariogenic acid attacks over a longer period of time [10]. NaF varnish was modified to incorporate amorphous calcium phosphate (ACP) formula. Unlike the former one, it delivers ACP to enamel to encourage the formation of hydroxyapatite (HAP) to enhance remineralization and thus prevents the loss of enamel due to demineralization [11]. On the other hand, fluoride-releasing, light-cured resin coat have additional advantages as they form a mechanical barrier between plaque and the enamel surface under and around orthodontic brackets which can be added before or after bracket bonding [12].

Another remineralization innovations derived from milk casein are casein phosphopeptide-stabilized amorphous calcium phosphate complexes (CPP-ACP) and casein phosphopeptide amorphous calcium phosphate with fluoride (CPP-ACFP). The main benefits of these materials attributed to their ability to localize at tooth surface and incorporate into supragingival plaque to provide bioavailable calcium (Ca) and phosphate (P) ions where they are most needed [13, 14].

Bleaching agents in varying concentrations have been used to achieve rapid esthetic results. Hydrogen peroxide and carbamide peroxide have been used successfully for many years to achieve lighter and more desirable tooth color [15, 16]. However, several deleterious effects of bleaching on enamel were reported. Among those effects are changes in micro hardness, changes in surface roughness, presence of porosities, alteration of Ca/P ratio, reduction in fracture toughness, erosion, and formation of depressions [17]. Therefore, it could be considered as a predisposing factor to WSLs formation around orthodontic brackets.

To the best of our knowledge, the previous studies focused mainly on the effect of bleaching on enamel surface roughness and the bond strength of orthodontic brackets [15–17]. In the orthodontic practice, little and insufficient data were found regarding the effect of bleaching on either enamel decalcification or the efficacy of the topical agents utilized for decalcification prevention of bleached enamel. Therefore, the present study was conducted to evaluate and compare five different topical agents utilized for prevention of demineralization around orthodontic brackets bonded to bleached and non-bleached enamel surfaces. The research null hypothesis was that no effect of bleaching on enamel decalcification and that no difference between the different topical agents on prevention of enamel decalcification around orthodontic brackets.

## Methods

One hundred and twenty recently extracted human maxillary premolars were utilized in this study. The sample size was estimated by G*Power (version 3.0.10, Kiel University, Germany). Assuming type I statistical error of 5% and two-tailed statistical test, this study was designed to have a power of 90% based on previous study [18]. The calculated sample size was 10 teeth per subgroup.

The teeth were selected according to the following criteria: intact buccal enamel surface, no extraction damage or pitting and cracks and free of caries, dental fluorosis, and other hypomineralized lesions. Investigating the buccal surface was done with the aid of eye loupes ×6 magnification (Univet, Italy). All teeth were washed with tap water and stored in 0.1% thymol solution. The teeth were randomly divided into two main equal groups. Group I (*n* = 60) was left without bleaching and group II (n = 60) was subjected to bleaching with 30% carbamide peroxide gel (Vivastyle, Ivoclar Vivadent AG, Schaan/Liechtenstein, USA), according to manufacturer’s instructions. At first, the enamel surfaces were cleaned with non-fluoridated pumice and rubber cup connected to low-speed hand piece for 10 s. The carfaces for 30 min and repeated for 3 days. After bleaching, the teeth were rinsed with tap water. Finally, fluoride (Fluor protector, Ivoclar Vivadent AG, Schaan/Liechtenstein, USA) was applied for 10 min.

Metal brackets (NANDA, Ortho Organizers, CA, USA) were bonded to the buccal surfaces of all teeth. The buccal enamel area subjected to etching and bonding procedures was equal and standard in all teeth. The enamel was etched for 30 s with 37% phosphoric acid gel (Super Etch, SDI Limited, Bayswater, Australia), rinsed with water for 10 s and dried with air for 5 s. Transbond XT primer (3M Unitek, CA, USA) was applied to the etched enamel. Transbond XT adhesive (3M Unitek) was placed on the bracket base. The brackets were positioned on the correct position with firm pressure and excess composite was removed. Finally, the adhesive was light cured for 20 s.

Then, the teeth in either group were subdivided into equal six subgroups (A, B, C, D, E, and F) according to the materials utilized for prevention of enamel decalcification. All the materials were used according to manufacturer’s instructions.

*Subgroup A*: no material was applied (control).

*Subgroup B*: 5% NaF varnish (Profluorid varnish, VOCO GmbH, Cuxhaven, Germany) was applied on the labial surfaces around the brackets. Then, the teeth were leaved to dry for 5 min.

*Subgroup C*: 5% NaF-ACP varnish (Enamel Pro Varnish, Premier dental, PA, USA). The varnish was applied as in group B.

*Subgroup D*: fluoride-releasing, light-cured resin coat (Ortho-Choice Ortho-Coat, Pulpdent, Watertown, MA, USA) was applied. The coat was light-cured for 20 seconds.

*Subgroup E*: CPP-ACP (GC Tooth Mousse, GC Corp, Tokyo, Japan) was painted and left for 3 min undisturbed. Then, it was removed by cotton roll and allowed to dry for extra 2 min. Finally, the remaining residue was washed with tap water. These procedures were repeated daily.

*Subgroup F:* the same procedures were done as in subgroup E except CPP-ACFP (GC MI Paste plus, GC Corp, Tokyo, Japan) was applied instead of casein phosphopeptide amorphous calcium phosphate complex.

All teeth were cycled in a demineralization solution and artificial saliva for 15 days [18]. The teeth were immersed in the demineralizing solution (calcium nitrate 0.4723 g, potassium dihydrogen phosphate 0.2722 g, acetic acid 4.5083 g) for 8 h/day [19]. Then, the teeth were rinsed with tap water and putted in artificial saliva solution (sodium azide 0.75 g, potassium monohydrogen phosphate 0.804 g, sodium chloride 1.02 g, calcium chloride 0.166 g, magnesium chloride 0.059 g) for 30 min [20]. Each tooth was cleaned by using electronic dental brush (Oral-B, Braun GmbH, Germany) and Crest cavity protection toothpaste (1450 ppm F) for 2 s. Again, all teeth were rinsed with water and placed in the artificial saliva for approximately 15 h until the cycle was repeated.

Laser fluorescence device (Kavo DIAGNOdent pen, Kavo, Germany) was used to measure the level of mineralization of the enamel surfaces around the brackets. Measurements were taken before application of the materials utilized for prevention of enamel decalcification and after cycling in the demineralization solution and artificial saliva. At first, the laser device was calibrated against the device own ceramic standard to ensure an accurate reading. Then, measurements were obtained on the buccal surfaces 1 mm away and opposite the center of the mesial, distal, gingival, and occlusal borders of the brackets. The laser device gives readings from 0 to 99. Low readings indicate high mineral content of the enamel and hence clean healthy tooth structure. On the other hand, higher readings indicate greater demineralization.

The data were collected and analyzed using Statistical Package for the Social Sciences (SPSS version 17.0). Kruskal-Wallis test was used to evaluate if there was a significant difference in the changes between all subgroups. Mann-Whitney *U* test was utilized to compare the changes between each two subgroups. Wilcoxon signed-rank test was used to compare between the pre and post measurements in each studied subgroups. The statistical tests were based on a type 1 error value of 5% (*α* = 0.05) and on a power of 0.90 sample size.

## Results

### Evaluation of the measurements of non-bleached studied group

The median values of bleached and non-bleached studied subgroups are presented in Table 1 including the pre, post, and pre-post demineralization changes. The latter was calculated statistically by subtracting the pre demineralization value from the post demineralization one. Regarding the non-bleached subgroups, the control one showed the highest value of demineralization change (22.5) followed by GC Tooth Mousse (4.5) and GC MI Paste plus (2.5). No demineralization changes were observed in either Profluorid varnish or Enamel Pro Varnish subgroups. In contrast, Ortho-Choice Ortho-Coat subgroup showed remineralization change (−1).

Kruskal-Wallis test revealed a significant difference between the changes in non-bleached studied subgroups (*P* < 0.05). Furthermore, the results of Mann-Whitney *U* test (Table 2) revealed significant differences between all subgroups (*P* < 0.05) except between Profluorid varnish and Enamel Pro Varnish, Enamel Pro Varnish and Ortho-Choice Ortho-Coat, and GC Tooth Mousse and GC MI Paste plus subgroups (*P* > 0.05).

**Table 1:** Medians of DIAGNOdent values for different bleached and non-bleached subgroups

	Non-bleached group			Bleached group		
Subgroups	Pre demineralization	Post demineralization	Changes	Pre demineralization	Post demineralization	Changes
Control	4.0	26.50	22.5	3.0	4.50	1.0
Profluorid varnish	4.0	4.0	0.00	3.0	2.0	1.0-
Enamel Pro Varnish	3.0	3.0	0.00	2.0	2.0	1.0-
Ortho-Choice Ortho-Coat	5.50	4.0	1.00-	3.00	2.50	0.50-
GC Tooth Mousse	5.0	10.0	4.50	2.0	3.50	1.0
GC MI Paste plus	5.0	8.0	2.50	2.0	4.00	1.0

**Table 2:** Medians of DIAGNOdent values of demineralization changes for different bleached and non-bleached subgroups

Subgroups	Non-bleached group	Bleached group
	Changes	Changes
Control	22.5	1.0 ^A^
Profluorid varnish	0.00 ^A^	1.0- ^B^
Enamel Pro Varnish	0.00 ^AB^	1.0 ^BCD^-
Ortho-Choice Ortho-Coat	1.00- ^B^	0.50- ^BCD^
GC Tooth Mousse	4.50 ^C^	1.0 ^A^
GC MI Paste plus	2.50 ^C^	1.0 ^A^

Medians changes with the same superscript letters in the same column are non-significant according to Mann-Whitney U test. ( Significant at *P*<0.05)

Wilcoxon signed-rank test (Table 3) showed significant differences between pre and post demineralization cycle measurements in control, GC Tooth Mousse, GC MI Paste plus, and Ortho-Choice Ortho-Coat subgroups (*P* < 0.05). However, the changes that occurred in the former three subgroups were demineralization while it was remineralization in Ortho-Choice Ortho-Coat subgroup. On the other hand, no significant differences were found between pre and post demineralization cycle measurements in either Profluorid varnish or Enamel Pro Varnish subgroups (*P* > 0.05).

**Table 3:** Medians of pre and post demineralization DIAGNOdent values for bleached and non-bleached subgroups

	Non-bleached group				Bleached group			
Subgroups	Pre demineralization	Post demineralization	Z	*P* value	Pre demineralization	Post demineralization	Z	*P* value
Control	4.0	26.50	2.807-	0.005	3.0	4.50	-2.555	0.011
Profluorid varnish	4.0	4.0	0.137-	0.891	3.0	2.0	-2.414	0.016
Enamel Pro Varnish	3.0	3.0	1.473-	0.141	2.0	2.0	-2.530	0.011
Ortho-Choice Ortho-Coat	5.50	4.0	2.401-	0.016	3.00	2.50	-1.667	0.096
GC Tooth Mousse	5.0	10.0	2.807-	0.005	2.0	3.50	-2.825	0.005
GC MI Paste plus	5.0	8.0	2.405-	0.016	2.0	4.00	-2.724	0.006

Significant at *P*<0.05

### Evaluation of the measurements in bleached studied group

The medians of bleached studied subgroups are presented in Table 1. Demineralization changes were found in the control, GC Tooth Mousse, and GC MI Paste plus subgroups. Remineralization changes were observed in Profluorid varnish (−1), Enamel Pro Varnish (−1), and Ortho-Choice Ortho-Coat (−0.5) subgroups.

The results of Kruskal-Wallis test revealed a significant difference between the changes in bleached studied subgroups (*P* < 0.05). In addition, the results of Mann-Whitney *U* test (Table 2) revealed significant differences between all subgroups (*P* < 0.05) except between control and GC Tooth Mousse, control and GC MI Paste plus, Profluorid varnish and Enamel Pro Varnish, Profluorid varnish and Ortho-Choice Ortho-Coat, and Enamel Pro Varnish and Ortho-Choice Ortho-Coat subgroups (*P* > 0.05).

Wilcoxon signed-rank test (Table 3) revealed significant differences (*P* < 0.05) between pre and post demineralization cycle measurements in all subgroups except in Ortho-Choice Ortho-Coat subgroup (*P* = 0.096). However, there was demineralization in control, GC Tooth Mousse, and GC MI Paste plus subgroups while there were remineralization in the remaining subgroups.

### Comparison between the demineralization changes for bleached and non-bleached subgroups

The demineralization changes between bleached and non-bleached subgroups (Table 4) were significantly different in control and Profluorid varnish and GC Tooth Mousse subgroups (*P* < 0.05). However, no significant differences were found in Enamel Pro Varnish, Ortho-Choice Ortho-Coat, and GC MI Paste plus subgroups (*P* > 0.05).

**Table 4:** Medians of DIAGNOdent values of demineralization changes for different bleached and non-bleached subgroups

	Group				
Subgroups	Non-bleached	Bleached	Mann-Whitney U	Z	*P* value
Control	22.50	1.00	0.000	-3.800	0.000
Profluorid varnish	0.00	-1.00	25.000	-1.982	0.047
Enamel Pro Varnish	0.00	-1.00	38.000	-0.967	0.334
Ortho-Choice Ortho-Coat	-1.0	-0.50	31.500	-1.467	0.142
GC Tooth Mousse	4.5	1.00	3.500	-3.562	0.000
GC MI Paste plus	2.5	1.00	27.000	-1.757	0.079

Significant at *P*<0.05

## Discussion

Development of WSLs around orthodontic brackets is still a common occurrence, jeopardizing the health and esthetics of the teeth [21]. Five different topical agents utilized for prevention of enamel demineralization was evaluated in the present study. The study was conducted on bleached and non-bleached enamel tooth surfaces.

Several methods have been used to determine enamel demineralization such as visual inspection, photographic examination, fluorescent dye uptake, ultraviolet light, and laser fluorescence [18, 22, 23]. The latter (DIAGNOdent pen) was used in this study. It is easy to use and a reproducible method. Also, it was proved to be an effective tool for decalcification evaluation. Shi et al. reported that DIAGNOdent has a high specificity for lesions in the outer half of the enamel [22]. Furthermore, Benham et al. showed that the DIAGNOdent was more accurate than visual assessment in detecting demineralization on the teeth [23].

According to the results of non-bleached subgroups, all studied materials provided significant resistance to demineralization. This result was consistent with the results of other authors [13, 18, 24–27].

Ortho-Choice Ortho-Coat provided the highest significant resistant to demineralization among the studied materials. This finding is in agreement with those of Hu and Featherstone, Salar et al., and Leizer et al., [26–28]. This probably attributed to the ability of Ortho-Choice Ortho-Coat to fluoride releasing which encourages the formation of CaF_2_ and fluorapatite that enhance remineralization of the enamel [29]. In addition, Ortho-Choice Ortho-Coat acts as a barrier around the bracket which may prevent the entry of saliva and oral fluid beneath the bracket and it provides smooth surface which prevents accumulation of dental plaque [12].

The results of present study also revealed that both Profluorid varnish and Enamel ProVarnish had moderate effects among the studied materials in preventing the demineralization around the brackets. These findings are in agreement with those of Nalbantgil et al., Vivaldi- Rodriques et al., Ulkur et al., and Farhadian et al., [10, 24, 25, 30]. Furthermore, no significant difference was found between the two varnishes although they have a different mechanism of action. The demineralization inhibitory effect of the Enamel Pro was attributed to the formation of ACP crystals and apatite on enamel surface [31]. On the other hand, Profluorid varnish produce deposits of CaF_2_ and depositing F in porosities and micro channel in enamel surface [32].

The least effect on reduction of demineralization was observed with utilization of GC MI Paste plus and GC Tooth Mousse, respectively. This result was in line with those of Behnan et al. [18]. The little effect of GC Tooth Mousse and GC MI Paste plus on prevention of enamel demineralization could be explained by a long-term application that may be needed to provide the desired effect (release of ACP from CPP and deposition of Ca and P into the enamel surface) [33]. The effect of GC MI Paste plus was significantly greater than that of GC Tooth Mousse. This could be contributed to the fluoride content of GC MI Paste plus augmenting its effect. This finding was in agreement with the results of Shetty et al., [34]. On other hand, our result was in disagreement with the results of Lata et al., who found that no clinical evidence effect on enamel remineralization by adding fluoride to CPP-ACP [14].

The results for bleached subgroups showed that both varnishes and Ortho-Choice Ortho-Coat provided significant resistance to enamel demineralization. No significant difference was found between these three materials. However, in contrast to non-bleached subgroups either GC Tooth Mousse or GC MI Paste plus did not significantly reduce enamel demineralization compared to the control. These could be attributed to fluoride application during bleached protocol may enhance mineralization of enamel surfaces which was manifested by the lower reading of DIAGNOdent in the bleached subgroups in comparison to the non-bleached subgroups. This enhancement significantly increased the demineralization reduction potential of the varnishes and Ortho-Choice Ortho-Coat. On the other hand, it did not significantly augment the effects of the caseins.

## Conclusions

- All studied materials significantly reduced enamel demineralization.
- Ortho-Choice Ortho-Coat had the highest potential to decrease enamel demineralization followed by Profluorid varnish, Enamel ProVarnish, GC MI Paste plus, and GC Tooth Mousse respectively.
- Either GC Tooth Mousse or GC MI Paste plus had no pronounced effects on prevention of enamel demineralization around orthodontic brackets bonded to bleached enamel surfaces.
